# Improvement on the Fatigue Performance of 2024-T4 Alloy by Synergistic Coating Technology

**DOI:** 10.3390/ma7053533

**Published:** 2014-05-06

**Authors:** Xi-Shu Wang, Xing-Wu Guo, Xu-Dong Li, Dong-Yun Ge

**Affiliations:** 1 Department of Engineering Mechanics, Key Laboratory of Applied Mechanics, Tsinghua University, Beijing 100084, China; E-Mails: xdli236415064@gmail.com (X.-D.L.); gedy@tsinghua.edu.cn (D.-Y.G.); 2 National Engineering Research Center for Light Alloy Net Forming, School of Material Science & Engineering, Shanghai Jiao Tong University, Shanghai 200030, China; E-Mail: xingwuguo@sjtu.edu.cn; 3 Department of Mechanical Engineering, Qingdao Campus of Naval Aeronautical Academy, Qingdao 266041, Shandong, China

**Keywords:** plasma electrolytic oxidation, hard anodized, fatigue performance, synergistic coating, aluminum alloy

## Abstract

In this paper, rotating bending fatigue tests of 2024-T4 Al alloy with different oxide coatings were carried out. Compared to the uncoated and previously reported oxide coatings of aluminum alloys, the fatigue strength is able to be enhanced by using a novel oxide coating with sealing pore technology. These results indicate that the better the coating surface quality is, the more excellent the fatigue performance under rotating bending fatigue loading is. The improvement on the fatigue performance is mainly because the fatigue crack initiation and the early stage of fatigue crack growth at the coating layer can be delayed after PEO coating with pore sealing. Therefore, it is a so-called synergistic coating technology for various uses, including welding thermal cracks and filling micro-pores. The effects of different oxide coatings on surface hardness, compressive residual stress, morphology and fatigue fracture morphology are discussed. A critical compressive residual stress of about 95–100 MPa is proposed.

## 1. Introduction

The light alloys, such as aluminum and magnesium alloys, have been widely used in the automotive and aeronautic industries, due to their low density and high strength-to-weight ratio. In these fields, the general comprehensive requirements for the components include the high fatigue strength, good wear resistance and better corrosion resistance. That is, the first requirement is usually achieved by a proper material selection, which is accompanied by a high tensile strength and a higher cost [1–3]. The second requirement for the light alloys is achieved by using ceramic coating technologies with a high wear resistance [4,5]. Additionally, the third requirement for light alloys is usually achieved also by the selection of various coatings to avoid the corrosion of the substrate. Most research in the last ten years has indicated that the fatigue performances of most coated substrates were usually reduced compared with that of uncoated substrate, although there was a probability of either high wear resistance or excellent corrosion resistance [5–9]. For example, a hard anodized ceramic coating led to about a 75% reduction of the fatigue strength of 7475-T6 Al alloy, and a plasma electrolytic oxidation (PEO) coating resulted in about a 58% reduction of the fatigue strength of 7475-T6 Al alloy compared with the fatigue strength of the uncoated substrate [5]. In general, an anodized coating process is a classical approach to improve the tribological properties of light alloys. The relatively new surface engineering discipline of plasma electrolysis, such as PEO and plasma electrolytic saturation (PES), has triggered substantial research efforts focused on the applied surface science issues of light alloys during the last decade. The operation at low temperature, close to zero, allows the formation of relatively thick (up to 500 μm) and hard (up to 23 GPa) surface coating layers with excellent adhesion to either aluminum or steel substrate [10,11]. However, the various oxide coatings on the light metals have always a very low fatigue resistance or poor physical/mechanical performance compared with the uncoated substrate [5,12]. That is because the brittle property of the oxide coating layer, the connatural thermal-cracks in the oxide coating layer and either surface or internal defects, such as either pores or pits in the oxide coating layer, are sensitive to and induce a serious stress concentration under the applied loadings, especially under rotating bending or plane bending loadings, in which there is a stress gradient from the surface to the internal layer. Although the tribological properties or high corrosive resistance of these oxide coatings have been extensively studied in the last decade [11–14], there is relatively seldom any information available on the improvement of the fatigue behaviors of light alloys, either aluminum alloys or magnesium alloys, with an oxide coating. For example, it was reported [13] that the PEO coating might also cause no more than a 10% reduction in the fatigue limit of magnesium alloys. One of the most important factors influencing the fatigue strength of the PEO coating was thought to be the internal residual stress. Khan *et al.* [14] have previously characterized the residual stress attributed to the α-Al_2_O_3_ constituent of an 11.6 μm-thick PEO coating on 6082 aluminum alloy. They found that the residual stresses were of a compressive nature. However, it is recognized that the compressive residual stress will retard fatigue crack initiation in the coating. Therefore, the fatigue life of the coated components should be increased [14,15] if the compressive residual stress in the coating layer has a reasonable value. When the compressive residual stress is too high (such as being over 800 MPa [5]), the delaminating or cracking of the coating layer may easily cause the degradation of fatigue performance for most aluminum alloys. Especially, when the thickness of the oxide ceramic coating is over 100 μm, either surface multi-cracks or interface cracks may be induced during the applied loading process [16–19]. Therefore, one of the aims of this work is to search for a method for PEO coating technology while, or as closely as possible, satisfying the two requirements mentioned above, especially to improve the fatigue performance of the aluminum alloy with the coating compared with that which is uncoated. Additionally, the other aim is to estimate the critical compressive residual stress value to assist in improving the fatigue strength of the coating/substrate structure.

## 2. Experimental and Material

**Figure 1.**
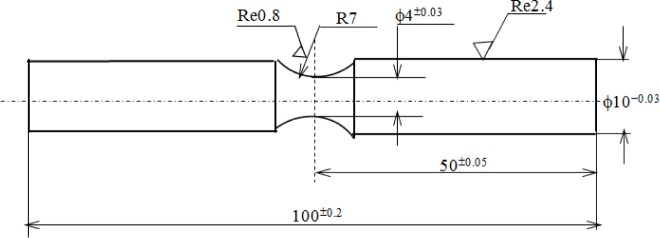
Shape and size sketch of the specimen; all dimensions’ unit: mm.

The present study has been conducted on 2024-T4 aluminum alloy containing a 0.6% Mn, 0.8% Si, 1.5% Mg, 4.4% Cu and Al balance. The main mechanical properties of 2024-T4 aluminum alloy are a yield stress (0.2% offset) of σ_0.2_ = 325 MPa, a tensile strength of σ_b_ = 470 MPa, an elongation of δ = 20% and a Young modulus of 69 GPa, respectively. A circular center notch of each specimen was machined in order to control the stress concentration factor as about 1.08, as shown in Figure 1, in which the main geometrical parameters are that the radius of the notch is 7 mm and the minimum diameter is 4 mm, respectively. All specimens were shaped by turning and grinding to the required dimensions and then polished by abrasive paper (1000) to achieve a surface roughness of approximately 0.2 μm *R*_a_ prior to three anodized coatings processes. Subsequently, the applied stress amplitude in these rotating bending fatigue tests to be estimated is as follows:

$$\sigma =\frac{32\text{g}\alpha LW}{\pi d^{3}}(\text{MPa})$$ (1)

where *d* is the diameter of the critical section (*i.e*., 4 mm), g is the acceleration of gravity (9.8 m/s^2^), α is the stress concentration factor (1.08), *L* is the distance from the critical section coming to the loading end (40.5 mm for a standard sample) and *W* is the applied loading (9.8N), respectively. All rotating bending fatigue tests were controlled by the load at a stress ratio *R* = −1 and in a rotating frequency of 50 Hz according to ASTM E468-90 in ambient atmospheric conditions [20].

The three typical coatings were produced by: (1) hard anodized coating; (2) plasma electrolytic oxidation (PEO) coating; and (3) PEO coating with impregnation of epoxy resin deposited on the same substrate of 2024-T4 aluminum alloy, respectively. The hard anodized coating was processed in the main parameters, such as a current density of 3.0 A/dm^2^ and a temperature of 0.0 ± 1.0 °C, according to the literature [5]. Additionally, the main steps of the PEO process are summarized as follows: (1) degreasing 2024-T4 aluminum alloy at 65 °C for 5–10 min; then rinsing in pure water and drying in the air for 1–2 min; (2) the PEO electrolytic solution consists of an alkaline caustic, phosphate- and silicate-diluted solution with a pH value of about 9; (3) a cooling system to maintain the electrolyte temperature below 30 °C is used; (4) the current density of PEO is 10 A/dm^2^; the pulse frequency is 1000 Hz; the time of oxidation is 15 min; and (5) the step of PEO with the impregnation of epoxy resin was carried out after the PEO process in which the surface micro-pores or micro-cracks of coating surface after the PEO process were sealed by means of electrode position with epoxy resin, and the PEO coating with the impregnation of epoxy resin layer was prepared.

**Figure 2.**
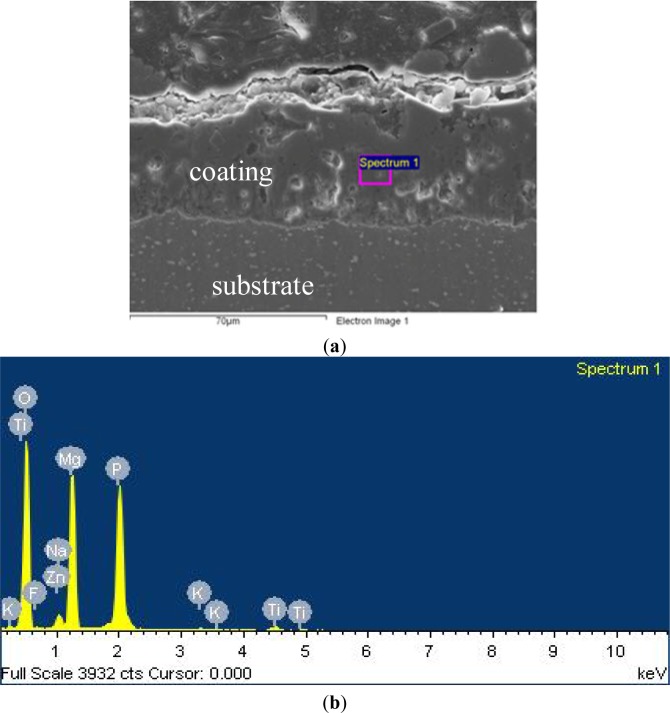
Spectrum process. (**a**) Cross-section of PEO coating; (**b**) spectrum peaks.

The compositions of the PEO coating are shown in Figure 2 and Table 1, respectively. The good interface adhesion between the coating layer and substrate is clearly seen in the cross-section of the PEO coating layer, which depends fully on the degree of surface roughness of the substrate. Additionally, the thickness of the coating layer used in testing samples is about 80–100 μm for three typical anodized coatings.

## 3. Experimental Results

**Figure 3.**
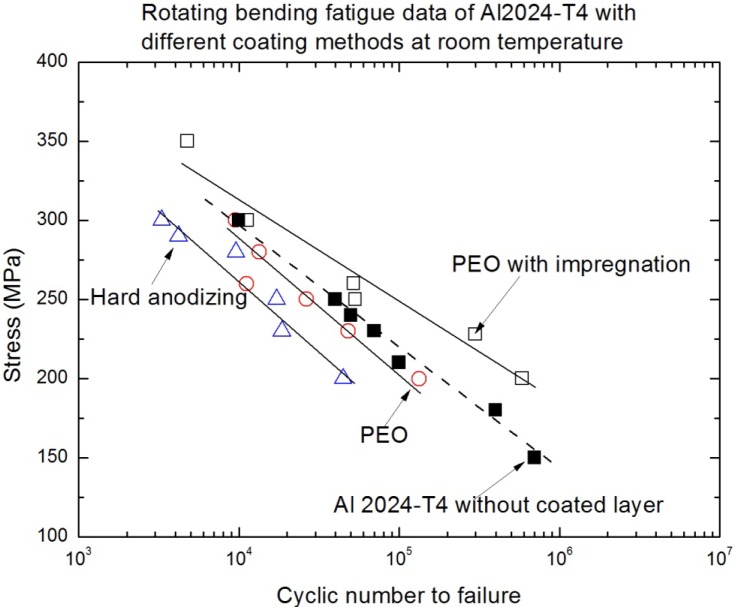
S-N curves of 2024-T4 Al alloy with the oxide coating and uncoated.

The dependence of the applied stress amplitude for these rotating bending tests, as shown in Equation (1), on the number of cycles to failure for the substrate (2024-T4 aluminum alloy) alone and the different oxide coatings on the same substrate were plotted in Figure 3. The S-N curves indicated that the fatigue strength of both hard anodized coated aluminum and PEO coated aluminum is lower than that of uncoated aluminum alloy. The stress as a function of the number of cycles to failure for the substrate alone and the coated substrates is similar to the changed tendency, as shown in the previously reported results [5,11–13]. The magnitude of fatigue strength reduction depends strongly on the stress amplitude during the rotating bending fatigue tests, and the reduction degree of the fatigue strength of 2024-T4 Al alloy with a hard anodized coating process is much more obvious than that with a PEO coating process and that with the uncoated substrate. With the increase in the stress amplitude, S-N curves tend to converge, thus reducing the negative effect of the coatings. This means that the effect of oxide coatings on the low cyclic fatigue of aluminum alloys is not obvious. That is because the low fatigue performance was caused by the brittleness of the oxide coating layer, in which the crack readily propagates to the substrate from the free surface [10]. Consequently, any crack that develops acts as a stress concentration level riser and will contribute to fatigue crack initiation and the early stage of fatigue crack growth. At the same time, the effect of the coating formed by the PEO process also slightly reduces the fatigue strength of the aluminum substrates, but the decrease in the fatigue strength of the PEO coating was much lower than that of the hard anodized coating, although the fatigue strength of the PEO coating is still smaller compared with the uncoated aluminum alloy. On the other hand, it can be clearly seen in Figure 3 that the fatigue strength is enhanced after PEO with the impregnation of epoxy resin compared with that of the uncoated 2024-T4 alloy and that after other oxide coating processes. The changed trends of S-N curves indicated the increasingly positive effects of the PEO coating/substrate structure with the decreasing of the stress amplitude. This hints that the improvement of the fatigue performance of light alloys through the PEO coating and appropriate impregnation treatment is possible. The S-N curves are able to quantitatively estimate that the reduction of the fatigue life that resulted from the hard anodized and PEO coating are about 70% and 25% compared with that of the uncoated substrate of 2024-T4 alloy, respectively. Additionally, the PEO coating with the impregnation of epoxy resin could enhance a high-cyclic fatigue (HCF) life (from 10^5^ to 10^6^) of about 50% compared with that of the uncoated substrate. At the same time, the variation tendency of S-N curves for the PEO coating with the impregnation of epoxy resin shows that the enhancing effect is also much more obvious with the decreasing of the applied stress level, *i.e*., it is less than 200 MPa, as shown in Figure 3. Therefore, the enhancing effect of PEO with the impregnation of epoxy resin on fatigue performance should have a much better result in the high-cyclic fatigue or giga-cyclic fatigue cases [21].

## 4. Analysis and Discussion

### 4.1. Effect of Typical PEO Coatings on Surface Hardness

The brittleness of the oxide coating layer is regarded as one of the important effective factors for causing fatigue crack initiation and the early stage fatigue crack growth behavior of light alloys after several thousand cycles. The average values of Webster hardness for the different PEO coating layers and uncoated 2024-T4 aluminum alloy are listed in Table 2. All hardness tests were carried out to be in accordance with the GB4340-84 standard (in China) as the number of iterations = 5 and the “M” array of indentations. These differences of three surface hardness average values in Table 2 are rather obvious. Although these differences of average hardness values do not include the average hardness value of the hard anodized coating, the previous reference had indicated the relative results between hard anodized coating and PEO coating [6]. In addition, synthetically considering the differences of the different S-N curves, as shown in Figure 3, it can be clearly seen that this reflects the fact that the lower the surface hardness for the oxide coating layer is, the stronger the fatigue strength of specimens is under rotating bending fatigue loading. The detected results of surface hardness indicate that the surface brittleness of the oxide coating contributes to the surface fatigue crack initiation and the early stage of fatigue crack growth, subsequently inducing the reduction of the fatigue crack initiation life of the coating/substrate structure. In addition, the stress at the free surface is the maximum stress in the cross-section of the sample in the rotating bending loading state, so that the fatigue crack initiation position occurs easily at the free surface. When a deformation mismatch occurs at the interface between the hard brittle oxide coating layer and the relatively softer aluminum substrate surface (and this easily occurs at the interface when the hardness difference is relatively high), the brittle oxide coating will be more easily delaminated compared with the relatively soft oxide coating. However, it is inferred that the interface adhesion strength of both PEO and PEO with the impregnation of epoxy resin is approximately the same, because the hole sealing treatment is only on the free surface or sub-surface in the coated layer, not influencing the interface adhesion. Therefore, the difference of fatigue life between the PEO coating and the PEO with the impregnation of epoxy resin is caused by the difference of the fatigue crack initiation life. Additionally, the difference of the fatigue life and influencing factors (except the surface hardness, without discussion) between the hard anodized coating and the PEO coating on the aluminum alloys has been described in previous literature [5–14]. The synergistic effects of oxide coating and substrate attracted widely studied interests, by means of choosing different processing parameters in the anodized coating process. In view of the mechanics or the damage mechanics, either enough of a higher capacity surface plastic deformation or the excellent surface quality in the oxide coating layer is able to improve the fatigue cracking resistance of the coating/substrate structure. Therefore, it is also not difficult to understand the trend change of the different S-N curves, as shown in Figure 3, according to the different hardness values corresponding to the fatigue strength of the different coating types and the uncoated aluminum alloy. One of the reasons for enhancing the fatigue life of the PEO coating with the impregnation of epoxy resin might be not only the “welding” of surface cracks, but also the filling of the micro-pores previously formed in the PEO coating process. This so-called synergistic effect between oxide coating and substrate can significantly improve the surface fatigue crack initiation and early stage fatigue crack growth resistance. Therefore, the fatigue strength of the PEO coating with the impregnation of epoxy resin is better than that of the other oxide coatings, so much so, that it is better than that of the uncoated substrate, as shown in Figure 3.

### 4.2. Effect of Different Oxide Coatings on Surface Morphology

**Figure 4.**
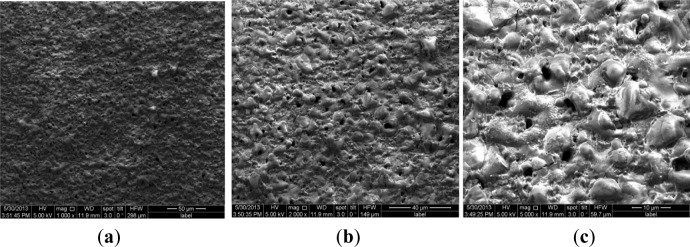
Typical surface morphologies of the PEO coating. (**a**) ×1000, scale bar 50 μm; (**b**) ×2000, scale bar 40 μm; (**c**) ×5000, scale bar 10 μm.

**Figure 5.**
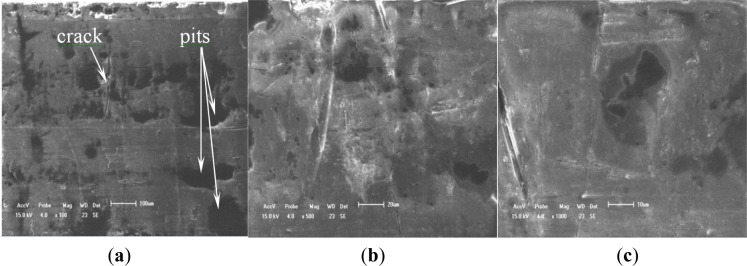
Typical surface morphologies of the hard anodized coating. (**a**) ×100, scale bar 100 μm; (**b**) ×500, scale bar 20 μm; (**c**) ×1000, scale bar 10 μm.

**Figure 6.**
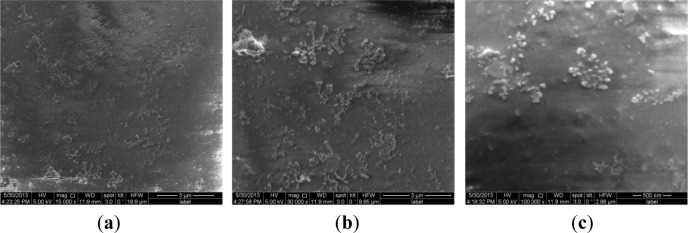
Typical surface morphologies of PEO coating with the pore sealing treatment. (**a**) ×15,000, scale bar 5 μm; (**b**) ×30,000, scale bar 3 μm; (**c**) ×100,000, scale bar 0.5 μm.

To validate the difference of free surface quality for the different coating processes, Figures 4 and 5 show their free surface morphologies. Due to the micro-arc surface discharges occurring throughout the coating penetrating through to the oxide deposition onto the 2024-T4 Al substrate, destructive effects, such as either thermal cracking or pores, were easily produced in the oxide coating layer. Therefore, the exterior or interior defects in the anodized coating layer are unavoidable. By means of comparison with the relative roughness and surface quality, including prior to thermal cracks and either micro-pores or meso-pits, the relative roughness of PEO with the impregnation of epoxy resin coating process is obviously better than that of both the single PEO coating and hard anodized coating processes, even though there is low conductivity of the Al_2_O_3_ coating by using the PEO coating with the pore sealing treatment. The original thermal cracks and micro-pores or meso-pits in the PEO caused by the pore sealing process are seldom seen, even at a scale bar of 500 nm, as shown in Figure 6a,c, but there are a lot of surface defects in other oxide coating layers, as shown in Figures 4 and 5, with the scale bar from 10 μm to 100 μm. The characterizations of the surface defects in the PEO coating process are simple to conclude: that there are relatively uniform shrinkage pores (the average diameter of the shrinkage pore is not over 10 μm) and a relatively rough surface, as shown in Figure 4b,c. Additionally, the characterizations of the surface defects in the hard anodized process are also simple to conclude: that there are heterogeneous pits (the maximum size of a pit is more than 100 μm, and the shape geometry of the pit is also not regular) and surface thermal cracks (the thermal crack length is over 100 μm), as shown in Figure 5a, but there is a relatively smooth surface, as shown in Figure 5b,c. In addition, there are other differences in the density and distribution of these defects, which are the pits and either pores or thermal cracks in both the hard anodized coating and the PEO coated surface. The main reason for the forming heterogeneous pits’ distribution is the difference between the thermal expansion coefficients of the aluminum alloy and the oxide coating layer [5,11,14]. These defects easily caused fatigue crack initiation, and the early stage of fatigue crack growth of aluminum alloys occurred at the free surface, as Li *et al.*, reported previously: similar pits (the maximum size of a surface corrosion pit is about 80–150 μm) can easily cause fatigue crack initiation and propagation [21]. The existence of the surface defects in the anodized coating layer can cause the reduction of the fatigue resistance of the anodized aluminum components. However, the appropriate surface treatment can also delay the fatigue crack initiation and the early stage of fatigue crack growth, such as the PEO with the impregnation of epoxy resin method. This is because fatigue crack initiation (high-cyclic fatigue) is strongly sensitive to a stress concentration influence, such as a notch, a microscopic defect [22], and the fatigue crack is also much more sensitive to the ductile property of the material, especially with a high brittleness of the surface of the oxide coating (Al_2_O_3_) material [23]. Therefore, it is not difficult to understand another important factor (surface quality or surface pattern) by which we decrease the fatigue strength of both the hard anodized coating and the PEO coating based on the morphology analysis among three oxidation coating/substrate structures. The correlation between fatigue damage and the state prior to thermal cracks, as well as either micro-pores or meso-pits in both the PEO coating surface and the hard anodized coating surface, as shown in Figures 4c and 5a, which are induced by the solidification and shrinkage of the molten aluminum oxide spots, of these surface defects can accelerate the fatigue damage of the coating/substrate structure under rotating bending cyclic loading, especially in high-cyclic fatigue damage (N_f_ ≥ 10^5^, as shown in Figure 3). These experimental data are in good agreement with those in the literature [3,5,6,9,21,24].

### 4.3. Effect of Different Oxide Coatings on Fatigue Fracture Morphology

**Figure 7.**
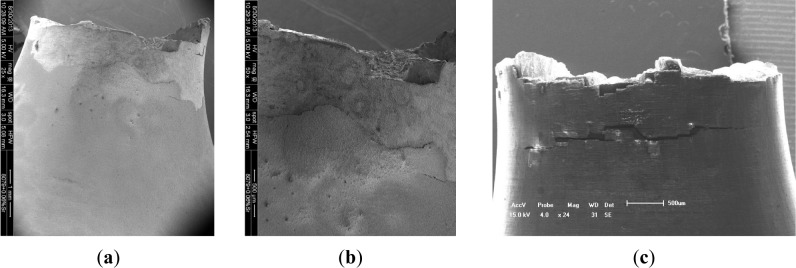
Typically macroscopical coating delaminating characterizations of PEO and hard coatings after fatigue tests. (**a**) PEO coating (scale bar: 1 mm); (**b**) PEO coating (scale bar: 0.5 mm); (**c**) hard anodized coating (scale bar: 0.5 mm).

In our previous works about the interface failure analysis of the film/coating-substrate structure [16–18], the film/coating failure model depended strongly on the cohesive strength, the residual stress in the inter-layer and the film/coating thickness, as well as the stress state. When plastic deformation existed on the free surface during fatigue tests, the delaminating or cracking behavior of the film/coating layer occurs easily in the surface or sub-surface of the film/coating-structure. Figure 7 illustrates the delaminating and cracking characterizations of anodized coatings. After rotating bending fatigue tests, the typical fracture surfaces of two types of coating samples were checked, in which there are differences as follows: (1) delaminating behavior is found on the surface of the PEO coating, as shown in Figure 7a,b; however, it is seldom found on the surface of the PEO with the impregnation of epoxy resin, due to the effective viscoelasticity action of the coating; (2) fatigue multi-cracks and small-sized delamination are found in the hard oxide coating process, as shown in Figure 7c.

One of the effective reasons may be the difference of compressive residual stress among oxide coating processes. For example, the average compressive residual stresses (in the surface or sub-surface) of the hard anodized coating, PEO coating and PEO with the impregnation of epoxy resin are 208.4 MPa, 109.5 MPa and 91.1 MPa, respectively. The results validated that it is correct that if the compressive residual stress is high, coating delamination and cracking may occur [4,5], degrading the fatigue properties of the coating-substrate structure. However, the appropriate compressive residual stress is able retard fatigue crack initiation in the coating; then, the fatigue life of the coated components increases [7–16]. Asquith *et al.* reported that the effect of combined shot peening and PEO treatment on the fatigue life of 2024-T3 Al alloy is an increasing of the fatigue limit when compared with aluminum treated only with PEO. Especially, the compression region with the maximum stress is about 160 MPa in the middle region of the coating layer [9]. Therefore, synthetically considering the effect of residual stress on the fatigue life of aluminum alloys, the critical value of the compressive residual stress might be deduced to be not over 95–100 MPa to enhance the fatigue life of 2024 Al alloy. This is because the compressive residual stress of the uncoated sample is zero, and the fatigue lives of the uncoated samples are less than that of the PEO coating with the impregnation of epoxy resin (σ_re_ = 91.1 MPa) and over that of the PEO coating alone (σ_re_ = 109.5 MPa).

**Figure 8.**
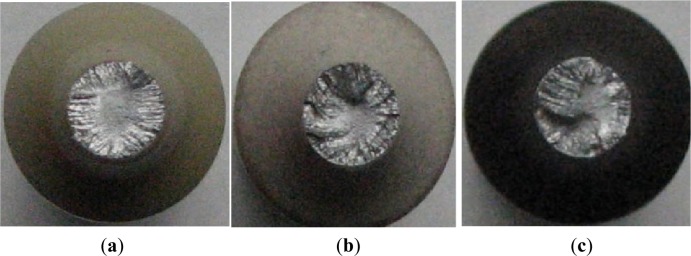
Fatigue fracture morphology for different oxide processes of 2024-T4 Al alloy (**a**) PEO with sealing pore; (**b**) PEO; (**c**) hard anodized.

The cross-section fracture surfaces of typical fatigue tested specimens were investigated by scanning electron microscope (SEM). Figure 8 illustrates the fatigue fracture morphology with the different oxidation coatings, including the PEO coating with the impregnation of epoxy resin, the PEO coating and the hard anodized coating. The fracture surfaces have much more different surfaces, such as the concave-convex regions or the different fatigue crack propagation regions from the marginal regions to the center, as shown in Figure 8a–c. This reflects the fact that the different surfaces indicate the different fatigue crack propagation lives (the fatigue life is usually defined as the fatigue crack initiation life and crack propagation life). That is, the fatigue crack propagation life of the PEO coating with the impregnation of epoxy resin is slower than that of the PEO coating and that of the hard anodized coating. This is because the center region and the fatigue crack propagation region in the marginal zone for the PEO coating ceramic with the impregnation of epoxy resin are smoother and larger than the other two oxidation processes. This means that the surface quality or different brittleness degrees (except for the effect of the residual stress) in the oxidation coating layer play an important part in the fatigue damage of aluminum alloy. The shorter the fatigue life is for the hard anodized coating process under the same applied stress, a reduction in the fatigue strength of the hard anodized samples was associated with cracking. The strong adhesion between coating and substrate allows the crack propagation from the coating through the interface into the substrate. In addition, the fracture-resistance of the different fracture appearances is different among the oxidation coating processes mentioned above. The different oxidation coating processes unavoidably cause different surface quality. Therefore, the oxidation coating with the impregnation of epoxy resin can much more easily avoid the surface defects and retard fatigue crack initiation. Additionally, the surface defect unavoidably causes the stress concentration and fatigue life degradation of materials. In addition, the synergistic effect of the PEO coating with the impregnation of epoxy resin is the best among the oxidation coating processes.

## 5. Conclusions

Based on the different oxide coatings, including the PEO coating alone, the PEO with the impregnation of epoxy resin coating and the hard anodized coating, the effects of different oxide coatings on the fatigue performance of 2024-T4 aluminum alloy were investigated. It was found that it is possible to enhance the fatigue performance of 2024-T4 aluminum alloy by using the surface treatment of the PEO coating with the impregnation of epoxy resin. The following conclusions were obtained:

1. The PEO coating with the impregnation of epoxy resin is not only “welding” the original thermal cracks, but also filling in either the micro-pores or meso-pits previously formed in the PEO coating process or the hard anodized process. This so-called synergistic effect between the coating and substrate can significantly improve the surface fatigue crack initiation resistance and the early stage of fatigue crack propagation resistance.
2. The S-N curves indicate that the oxide coating produced on 2024-T4 Al alloy by PEO with the impregnation of epoxy resin process has more excellent fatigue resistance than those obtained by hard anodized, PEO alone and uncoated substrate alloy. This can be attributed to the decrease of the stress concentration, the improvement of the surface quality of the coating and the retarding of fatigue crack initiation in the coating layer.
3. It has been found that the existence of either micro-pores or meso-pits and high compressive residual stress in the oxide coating layer can be believed to be the most important factors or dominant reasons for the fatigue strength reduction of the coating/substrate structure in the present work.
4. Compared with the surface and fatigue fracture morphology of different oxide coating processes, the surface quality of PEO with pore sealing is the best, and the fatigue crack initiation resistance is the greatest, as well as the fatigue crack propagation region being relatively uniform and constant.
5. The appropriate compressive residual stress is able to retard fatigue crack initiation in the coating, and therefore, the fatigue life of the coated components increases. The critical value of the compressive residual stress of the PEO coating 2024-T4 Al alloy might be deduced to be not over 95–100 MPa.

## Figures and Tables

**Table 1.** The data of the spectrum detection for the PEO coating (number of iterations = 3).

Element	Weight (%)	Atomic (%)	Standard
O K	53.13	66.17	O SiO_2_ 1 June 1999 12:00 AM
F K	1.40	1.47	F MgF_2_ 1 June 1999 12:00 AM
Na K	1.76	1.52	Na Albite 1 June 1999 12:00 AM
Mg K	18.56	15.21	Mg MgO 1 June 1999 12:00 AM
P K	22.76	14.64	P GaP 1 June 1999 12:00 AM
K K	0.37	0.19	K MAD-10 Feldspar 1 June 1999 12:00 AM
Zn K	0.42	0.13	Zn 1 June 1999 12:00 AM
Ti K	1.60	0.67	Ti 1 June 1999 12:00 AM
Totals	100.00	–	–

**Table 2.** Webster hardness under different surface treatment cases.

Surface treatment methods	Hardness values	Experimental condition
PEO	630.30 ± 92.90	$\text{HV}=\frac{2P\sin(\theta /2)}{d^{2}}$
PEO with impregnation	39.81 ± 8.82	
Uncoated substrate	279.46 ± 18.31	*P* = 5 kgf, θ = 136°, *t* = 30 s
